# Psychological safety mediates attendance and recovery-related outcomes within the Phoenix: a sober-active community

**DOI:** 10.3389/fpubh.2025.1458026

**Published:** 2025-03-21

**Authors:** Katie M. Heinrich, Brett Wyker, Beth Collinson, David Eddie, David Best, Jacquelyn Hillios

**Affiliations:** ^1^Department of Research and Evaluation, The Phoenix, Denver, CO, United States; ^2^Recovery Research Institute, Center for Addiction Medicine, Massachusetts General Hospital, Boston, MA, United States; ^3^Department of Psychiatry, Harvard Medical School, Boston, MA, United States; ^4^Centre for Addiction Recovery Research, Leeds Trinity University, Leeds, United Kingdom

**Keywords:** addiction recovery, substance use, alcohol use, peers, community-based organization, hope, empowerment, health

## Abstract

**Background:**

People in recovery from a substance use disorder often have difficulties forming pro-social relationships or accessing supportive communities. Providing psychological safety within recovery communities may be uniquely beneficial, yet psychological safety has mostly been studied among professional organizations and not among vulnerable populations. This program evaluation study examined associations between attendance, psychological safety, and retrospective recovery-related changes.

**Methods:**

Participants included 204 members of The Phoenix (13% response rate) who completed a survey that addressed recovery status and current perceptions of psychological safety, with hope, connection, empowerment, motivation to stay sober, mental health and physical health at 3-months and thinking back to baseline (i.e., “thentest”). Demographic information and attendance data were also collected.

**Results:**

Structural equation modeling found a good fit for the model (χ^2^ = 187.40, *p* = 0.23; RMSEA =0.049, GFI = 0.90, CFI = 0.98, SRMSR = 0.05.) and all path coefficients were statistically significant (*p* < 0.05). Participants’ perceived psychological safety fully mediated the relationship between attendance and recovery-related outcomes. Attendance was also directly and positively associated with physical health.

**Conclusion:**

Due to positive improvements in health and recovery-related outcomes mediated by psychological safety, results show benefits of attending events hosted by The Phoenix for those in recovery from substance use. Additional research should further validate the importance of psychological safety as a key mediator of the recovery process.

## 1 Introduction

In 2019, substance use disorder (SUD) was reported to affect 20.4 million Americans (1). Although abstinence is part of SUD recovery for many individuals (2), recovery is a journey rather than a destination and has been explained as improving multiple domains of wellness irrespective of ongoing substance involvement (3). Mutual-help organizations and peer services for SUD often emphasize the importance of forming relationships which are supportive of an individual’s recovery (4).

Peer support where individuals with lived experience and related knowledge assist one another can be particularly helpful, especially in terms of assisting individuals to shift from a ‘substance user’ social identity to a recovery-oriented social identity (5). This is beneficial for individuals experiencing ambivalence about changing substance use behaviors and/or having low self-efficacy (6). Support and encouragement from others are key factors that contribute to the development of self-efficacy and motivation to stay sober (7, 8), along with general beliefs that a person can access necessary resources to deal with challenges and/or achieve their goals and aims (9). Self-efficacy leads to empowerment, which manifests by enabling individuals positively influence their own lives (10). Overall, sustained recovery has been linked with greater motivation to remain sober, perceived health status, levels of connection, self-efficacy, and empowerment (11–14).

Recovery is not merely stopping the use of substances, but rather a process of growth where new coping skills and healthy behaviors are adopted (15). Recovery is thought to emerge from hope, where a person believes in the possibility of a better future (13). To develop hope, vicarious experiences of observing others in recovery, persuasive communication about the benefits of recovery, and experiencing positive emotional responses (i.e., psychological attachment) after trying recovery, are likely beneficial (7). Hope can help foster motivation for goal-oriented behavior including connecting with others (13). How a person conceptualizes their interpersonal connections and interacts with others in recovery, also influences self-efficacy for maintaining recovery (16).

Successfully connecting individuals seeking SUD recovery to a community of pro-recovery peers improves outcomes, and results in improved quality of life (8, 11, 17). Without the formation of pro-recovery networks that are supportive of an individual’s recovery journey, individuals are more likely to experience social isolation, or a return to individuals and groups that engage in substance use, placing individuals at a heightened risk of relapse (18). As such, there is a pressing need for easily accessible SUD recovery support services that foster the development of pro-recovery relationships and engagement in environments that are supportive and conducive to personal growth and well-being (19).

Of potential importance in these environments, psychological safety has been described as a state in which individuals feel comfortable being open with others without fear of negative consequences (20). When individuals have trusting and supportive relationships, they are more likely to feel psychologically safe (21). The concept of psychological safety has predominantly been applied at an organizational level (22), with multiple studies exploring the positive impact of psychological safety among healthcare professionals (23). A key finding of this work is that when healthcare professionals feel psychologically safe, they are more likely to raise legitimate concerns that ultimately lead to improvements in quality of care (24). In their organization’s quest to develop perfect teams, Google identified that psychological safety was critical as it allowed workers to be fully present, share fears, and have difficult conversations (25), perhaps allowing for more meaningful connections. To our knowledge the concept of psychological safety has not been applied to the individuals receiving a ‘service’ per se (e.g., a group of individuals participating in social events together).

Psychological safety among work teams has previously been measured using seven psychological safety items developed and validated by Edmondson. Items capture shared perceptions among team members as to whether they believe others will not reject members for being themselves or stating what they think; the extent to which team members care about each other; if team members have positive intentions toward one another; and if team members respect the competence of others. Several studies measuring individually held perceptions of psychological safety within and among organizations have adapted this measure (26–29). For this study, we developed an organization-specific psychological safety measure (see methods section) that was based upon the items from Edmondson.

Psychological safety is considered a key part of trauma-informed services (30). Psychological safety is important both for service providers and survivors of trauma (30). Of note, physical safety is not sufficient for psychological safety, although it is an important component (30). When working with individuals who have experienced adverse childhood experiences, it is important to consider how environmental components (e.g., smells, sounds, persons) can be unexpectedly triggering (30), which can be addressed by developing physical and psychological safety via trustworthiness (31).

One organization working to provide physically and psychologically safe spaces is The Phoenix, a sober, active community, offering free meaningful social activities to anyone with 48 h of continuous sobriety from non-prescription substances. The Phoenix has emerged as a novel form of recovery support across North America and further afield. Most events are led by volunteers with direct or indirect lived experiences of SUD recovery, or individuals with a connection to The Phoenix’s mission, which is, “to build sober active community that fuels resilience and harnesses the transformational power of connection so that together we rise, recover and live.”^1^

**Figure 1:**
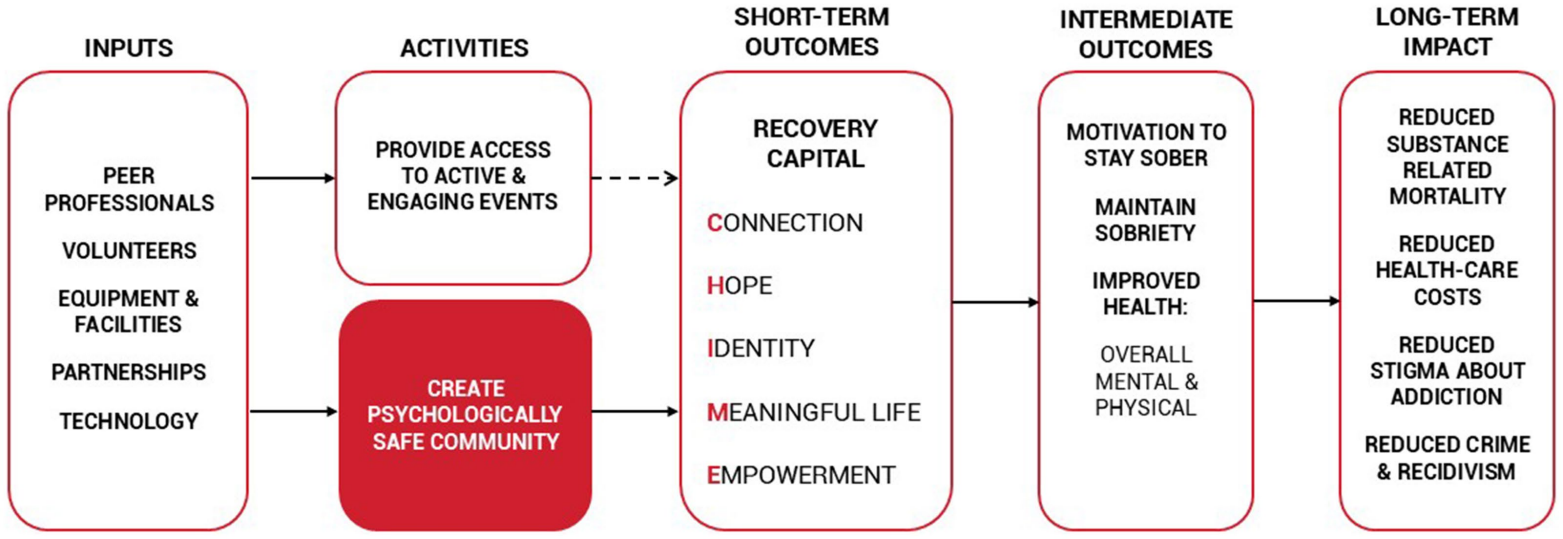
The Phoenix sober-active community model (58).

Fostering psychological safety is a key component of The Phoenix’s conceptual model (Figure 1), which is theorized to facilitate key short-term and intermediate outcomes that can have long-term impacts. To date, fostering psychological safety and relationships shown within the model have not been empirically tested.

The purpose of this study was to explore associations between attendance at Phoenix events, psychological safety, and recovery-related changes among Phoenix members. Specifically, the study aimed to identify, (1) if attendance at Phoenix events was associated with changes in intermediate outcomes changes (i.e., improved motivation to remain sober, mental health, and physical health); (2) if psychological safety mediated changes in the short-term outcomes of hope, empowerment, and connection; and (3) if changes in short-term outcomes were associated with changes in intermediate outcomes (i.e., improved motivation to remain sober, mental health, and physical health). We hypothesized that participants would rate their hope, empowerment, connection, and short-term outcomes changes higher at 3-months than at baseline and that this would be associated with greater attendance and psychological safety.

## 2 Materials and methods

### 2.1 Study setting, design, and participants

The data this study draws upon were collected as part of The Phoenix’s ongoing program evaluation, with Phoenix members being emailed an outcomes survey 3 months after initiating Phoenix participation. The survey was given exemption for review by the Ethical and Independent Review Services’ Institutional Review Board (#18170–01; 15 Oct. 2018), and the data presented here were collected between 2018 and 2019 from Phoenix members who had participated for 3 months.

The Phoenix offers a variety of sober active events, such as group fitness classes, rock climbing, group hikes, and yoga, and aims to cultivate a sense of psychological safety through its community standards, which are part of the member agreement. These standards state that The Phoenix promotes physical and emotional safety through prohibiting violent or threatening behavior or language; racist, sexist, homophobic or otherwise inappropriate language or conduct; unwelcome advances, physical contact, and/or sexually suggestive speech or actions; and weapons. Every event for The Phoenix begins with a review of the community standards. In addition, staff members and volunteers undergo training on the mission, vision, and guiding principles of The Phoenix, along with how to create a culture of healing through the community standards with the goal of lifting one another up. These factors are thought to collectively help staff members and volunteers facilitate spaces which are nurturing and promote healing.

This survey used a “thentest” design (32, 33) by asking members to report both currently, and to think back to when they initiated Phoenix participation. Although change is conventionally measured via the difference between baseline (i.e., pretest) and follow-up (i.e., posttest) (33), using a retrospective pretest (i.e., thentest) is useful for detecting changes in internal standards (i.e., recalibration response shift) (34). In this design, the thentest is administered during follow-up, where it is asked at the same time as the posttest by asking participants to “think back to how they were doing at the start” to provide a retrospective answer (33). Calculation of the posttest minus thentest is used to represent the effect of time for shifts in responses (33). This approach differs from simply asking participants to report on retrospective change, or how their behavior has changed (i.e., has decreased, has not changed, has increased), which has shown inconsistencies compared to pretest-posttest differences (35). Using the thentest is subject to recall bias (32, 33), yet allows for an introspective process that allows the participant to consider how they used to be as compared to now. Previous research has recommended that using the thentest method for shorter time periods such as 3 or 6 months should increase recall accuracy (36).

### 2.2 Measures

All survey items were pilot tested, refined and validated using responses collected from a sample of members in 2016 and 2017. Included items met the following psychometric benchmarks: (1) internal consistency of scale, Cronbach’s Alpha ≥0.70; (2) item “difficulty,” or the mean/ # response ideally between 0.20 and 0.80; (3) item discrimination, Correlated Item Total Correlation ≥0.40; and (4) Factor loading >0.70 with items of the same domain on one factor.

Survey items included, but were not limited to, recovery status, perceptions of psychological safety experienced at The Phoenix events, motivation to stay sober, perceived health status, levels of connection, and empowerment. Participants were asked to report their perceived psychological safety at the time of the 3-month assessment only as they lacked exposure to psychological safety within Phoenix events at baseline (37). For the other survey items, participants were asked to rate themselves on these measures retrospectively, thinking back to their first involvement with The Phoenix (i.e., thentest), and again currently at 3-months. Scores were calculated for each item or scale by subtracting the thentest rating from the rating for 3 months. Demographic information and attendance were also collected via attendance records at activities and events hosted by The Phoenix.

#### 2.2.1 Recovery status

Participants were given the following definition of recovery: “Recovery refers to the process of improving your physical, psychological, and social well-being and health after having suffered from a substance use disorder.” They were then asked whether they considered themselves to be in recovery and if so, whether they were new to recovery or in long-term recovery from a SUD. At the time of this study, membership in The Phoenix was focused on helping individuals who were struggling with substance use, whether they felt like they were in recovery yet or not.

#### 2.2.2 Psychological safety

To measure perceptions of psychological safety, a new 5-item scale was developed, based on the Team Psychological Safety measure (20) (see Supplementary Table S1), that asked members to rate to what degree they experienced various aspects of psychological safety while participating in Phoenix activities and events. Members responded to a series of statements on a five-point scale endorsing their agreement or disagreement with each item, from “not at all” (1) to “completely” (5). Statements included: (1) “I feel welcomed by The Phoenix community,” (2) “I feel valued by The Phoenix community,” (3) “I feel accepted by The Phoenix community,” (4) “I feel comfortable sharing my emotions with The Phoenix community,” and (5) “If I were to relapse, I am confident that I can return to The Phoenix without being judged.” Items were averaged to create an overall score between 1 and 5.

#### 2.2.3 Empowerment

Three items from the New General Self-Efficacy Scale (38) were used with minor wording modifications (see Supplementary Table S1) to assess empowerment. The measure focuses on competence across a variety of situations and has been found to provide information about general self-efficacy with fewer items than other measures (39). Items were rated on a five-point scale, ranging from “not at all” (1) to “completely” (5) and averaged with a higher score indicating higher empowerment.

#### 2.2.4 Connection

Four items were used from the Social Connectedness Scale-Revised (40) Participants responded using a six-point scale of “strongly disagree” (1) to “strongly agree” (6). Items were averaged, with a higher score indicating stronger connection to others.

#### 2.2.5 Mental health, physical health and motivation to stay sober

Following the format of the widely-used single indicator of Self-Rated Health (41) response options for single item questions (i.e., “In general, how would you rate your __?”) mental health (42), physical health (43), and motivation to stay sober (44) ranged from “poor” (1) to “excellent” (5).

#### 2.2.6 Hope

Three items were used that had been developed and pilot tested by The Phoenix to evaluate how members’ views about living a sober life may change over time. Items included “There are a lot of activities I enjoy doing sober”; “I can have fun without using drugs or alcohol”; and “The things I most enjoy doing are things best done sober.” Response options were scored on a five-point scale, ranging from “not at all” (1) to “completely” (5). Items were averaged, with a higher score indicated greater hope.

### 2.3 Data analysis

Data were imported into R (45). As noted above, change scores were calculated by subtracting thentest ratings from 3-month posttest ratings. Data were checked for assumptions such as normality including skewness and kurtosis. Psychological safety was highly skewed, as respondents generally rated their perceptions of psychological safety in the Phoenix community positively (mean = 4.52/5, SD = 0.72). Also, the event attendance counts were skewed toward the lower end of the distribution (48% attended five or fewer events in their first 3 months) and the range of the distribution was wide (ranging from 1 to 63 events attended.) Consequently, the skewness and kurtosis values were outside of the acceptable values for a normal distribution.

Bivariate tests (independent *t*-tests) were used to assess whether there were differences in mean scores of psychological safety at 3 months and differences (retrospective change scores) in hope, empowerment, connection, motivation to stay sober, mental health and physical health across age, gender and sexual orientation demographic groups (46). One-way ANOVAs were used to assess differences in the mean scores by recovery status groups.

Using the theory-driven approach to evaluation of Adodokun and associates (47), structural equation modeling (SEM) was used for hypothesis testing to quantify associations between attendance, perceived psychological safety, short-term, and intermediate outcomes for Phoenix members, including the relationship between hope and motivation to stay sober, social connection and self-reported mental health, and self-efficacy and self-reported physical health. To address the issue of non-normality for some measures, bootstrapping was used in all SEM models for model evaluation. For the bootstrap analyses, 2000 bootstrap samples were taken and then used to estimate bias-corrected confidence intervals and *p* values as implemented by Lavaan package in R (48). A multivariate measurement model was generated using maximum likelihood solutions. Next, a full latent variable structural model following the structure of The Phoenix sober-active community model was tested using SEM to determine the relative contribution of psychological safety to hope, empowerment and connection and the subsequent contributions to motivation to stay sober, mental health and physical health. Due to significant bivariate differences, models were adjusted for recovery status where hope and physical health were endogenous variables. Indices used to assess SEM measurement and structural model fit included chi-square, root mean square error of approximation (RMSEA), traditional goodness of fit indices (GFI), comparative fit index (CFI) and the Standardized Root Mean Square Residual (SRMSR).

## 3 Results

Of the 2,267 individuals who were emailed the survey during the study period, 294 (13%) responded. Only those without missing values on questions related to the domains of interest were included in the Structural Equation Model (SEM) analyses (*n* = 204). Individuals with complete and incomplete data did not significantly differ in demographic or other characteristics (e.g., recovery status). Demographic characteristics are shown in Table 1.

**Table 1:** Characteristics of Phoenix members who participated in the survey (*N* = 204).

Variable	N (%)	M (SD) [Min-Max]
Age (years)		39.3 (11.6) [19–77]
Young adult (under age 35)	81 (39.7)	
Middle age or older adult (ages 35 and older)	118 (57.8)	
Missing	5 (2.5)	
Gender		
Female	84 (41.2)	
Male	113 (55.4)	
Other (e.g., Transgender, Other gender)	7 (3.5)	
Race		
American Indian or Alaska Native	5 (2.5)	
Black / African American	13 (6.4)	
Native Hawaiian or Other Pacific Islander	2 (1.0)	
White / Caucasian	150 (73.5)	
Mix/Multiple Races	13 (6.4)	
Other	10 (4.9)	
Prefer not to answer	11 (5.4)	
Ethnicity		
Hispanic	20 (9.8)	
Sexual orientation		
Bisexual	5 (2.5)	
Gay	16 (7.8)	
Heterosexual	160 (78.4)	
Lesbian	2 (1.0)	
Other	3 (1.5)	
Prefer not to answer	18 (8.8)	
Recovery status		
New to recovery	78 (38.2)	
In long-term recovery	93 (45.6)	
Considering recovery	3 (1.5)	
Prefer not to answer	30 (14.7)	
Events attended (number)	1,595	8.1 (9.9) [1–63]

Results comparing mean differences in psychological safety and between thentests and posttests for outcomes of interest by demographic groups are presented in Table 2. Those who were new to recovery were significantly more likely than those in long-term recovery to report increased ratings for hope, *f*(2, 202) = 3.5, *p* = 0.033. As such, recovery status was included as a covariate in SEM models where hope was an endogenous variable. Although on average, members endorsed high levels of psychological safety (Mean = 4.5, SD = 0.7; Range 1–5); demographic groups did not differ on this measure.

**Table 2:** Comparisons of psychological safety at 3 months and changes in recovery-related outcomes by demographic groups.

	N	Psychological safety rating M ± SD	Hope difference M ± SD	Empowerment difference M ± SD	Connection difference M ± SD	Motivation to Stay sober difference M ± SD	Mental health difference M ± SD	Physical health difference M ± SD
Full sample								
Cohen’s d (95% CI)		–	1.0 (0.8, 1.2)	1.0 (0.8, 1.2)	0.9 (0.7, 1.0)	0.8 (0.7, 1.0)	0.9 (0.7, 1.0)	0.9 (0.7, 1.0)
	204	4.5 ± 0.7	1.2 ± 1.2	1.0 ± 1.1	1.1 ± 1.3	1.0 ± 1.2	1.1 ± 1.2	0.9 ± 1.1
Age^a^								
Cohen’s d (95% CI)		0.1 (1–0.2, 0.4)	0.1 (−0.2, 0.4)	0.2 (−0.1, 0.5)	0.2 (−0.1, 0.5)	0.001 (−0.3, 0.3)	0.01 (−0.3, 0.3)	0.03 (−0.3, 0.3)
Young adult	81	4.6 ± 0.6	1.3 ± 1.3	1.2 ± 1.0	1.3 ± 1.4	1.0 ± 1.3	1.1 ± 1.3	0.9 ± 1.1
Middle age/Older adult	118	4.5 ± 0.8	1.2 ± 1.2	0.9 ± 1.1	1.0 ± 1.2	1.1 ± 1.2	1.1 ± 1.2	1.0 ± 1.1
Gender^b^								
Cohen’s d (95% CI)		0.2 (−0.5, 0.1)	0.2 (−0.1, 0.5)	0.1 (−0.2, 0.3)	0.04 (−0.3, 0.2)	0.1, (−0.4, 0.2)	0.2 (−0.3, 0.3)	0.02 (−0.1, 0.4)
Male	113	4.5 ± 0.8	1.3 ± 1.3	1.1 ± 1.1	1.1 ± 1.2	1.0 ± 1.2	1.1 ± 1.2	1.0 ± 1.2
Female	84	4.6 ± 0.6	1.1 ± 1.1	1.0 ± 0.9	1.1 ± 1.3	1.1 ± 1.3	1.1 ± 1.2	0.9 ± 1.0
Sexual orientation^c^								
Cohen’s d (95% CI)		0.4 (−0.01, 0.7)	0.02 (−0.4, 0.4)	0.1 (−0.4, 0.5)	0.03 (−0.4, 0.4)	0.2 (−0.6, 0.2)	0.3 (−0.6, 0.1)	0.3 (−0.7, 0.1)
Heterosexual	160	4.6 ± 0.7	1.2 ± 1.2	1.0 ± 1.0	1.1 ± 1.2	1.1 ± 1.2	1.2 ± 1.2	1.0 ± 1.1
LGBTQ	26	4.3 ± 0.8	1.2 ± 1.4	1.1 ± 1.1	1.1 ± 1.6	0.8 ± 1.5	0.8 ± 1.1	0.7 ± 1.0
Recovery status								
η_p_^2^ (95% CI)		0.003 (0.0, 0.3)	0.03 (0.0, 0.9)*	0.02 (0.0, 0.1)	0.001 (0.0, 0.01)	0.01 (0.0, 0.04)	0.01 (0.0, 0.1)	0.02 (0.0, 0.07)
New to recovery	77	4.5 ± 0.8	1.5 ± 1.2^d^	1.1 ± 0.9	1.1 ± 1.2	1.0 ± 1.3	1.2 ± 1.3	1.1 ± 1.2
In long-term recovery	92	4.6 ± 0.7	1.0 ± 1.2	0.9 ± 1.1	1.1 ± 1.4	1.0 ± 1.2	1.0 ± 1.2	0.8 ± 1.0
Other	33	4.5 ± 0.8	1.4 ± 1.1	1.3 ± 1.0	1.1 ± 1.1	1.2 ± 1.2	1.3 ± 1.3	1.2 ± 1.1

*p < 0.05.

a Does not include 5 participants who did not provide their age.

b Does not include 7 participants of other genders.

c Does not include 18 participants who chose not to answer the question.

d Significantly greater than those in long-term recovery. Other for recovery status represents those considering recovery or who chose not to answer.

**Figure 2:**
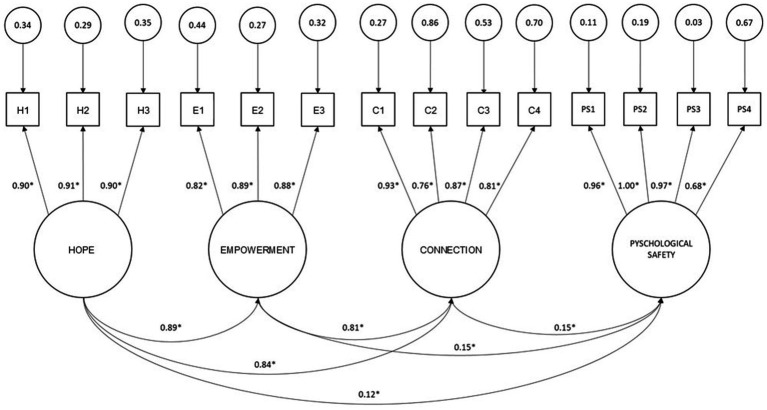
Measurement model standardized parameter estimates and latent variable correlations. H, Hope; E, Empowerment; C, Connection; PS, Psychological Safety.

A measurement model that included psychological safety, hope, connection, and empowerment measures was tested using SEM (see Figure 2). The measurement model was statistically overidentified. The Bollen-Stine bootstrap chi square test yielded statistically non-significant results (*χ*^2^ = 83.47, *p* = 0.42). The RMSEA was 0.03, the traditional GFI was 0.94, the CFI was 0.99 and the SRMSR was 0.048. The indices indicated a good model fit. All correlations between latent variables were statistically significant (*p* < 0.05).

**Figure 3:**
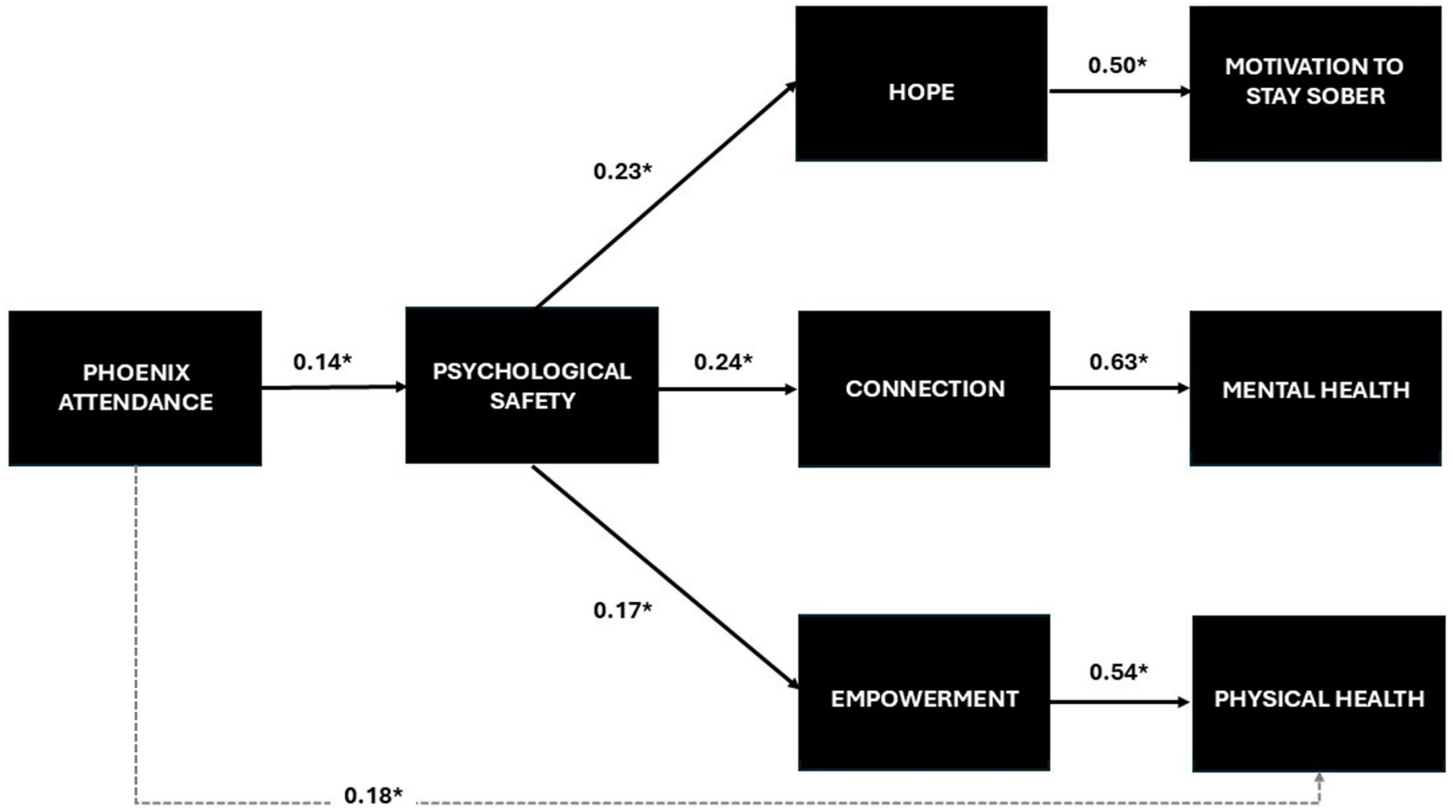
Full structural model and path coefficients testing the Phoenix conceptual model (*N* = 204).

A full structural model was tested using SEM, with attendance associated with psychological safety, psychological safety associated with changes in hope, connection, and empowerment; changes in hope associated with changes in motivation to stay sober; connection change associated with changes in mental health; and empowerment change (along with attendance directly) associated with changes in physical health. The model is presented in Figure 3, which includes the path coefficients generated from the analysis. For purposes of presentation, the correlation between exogenous variables and the recovery status covariate was omitted. All model fit indices pointed to good model fit (*χ^2^* = 187.40, *p* = 0.23; RMSEA = 0.049, GFI = 0.90, CFI = 0.98, SRMSR = 0.05.) All path coefficients were statistically significant (*p* < 0.05). The variables in the model explained 25% of variance for motivation to stay sober, 39% of the variance for mental health and 35% of the variance for physical health.

## 4 Discussion

We explored associations between attendance, psychological safety and retrospective recovery-related changes among members of The Phoenix using data collected as part of ongoing program evaluation efforts. We found significant relationships between Phoenix event attendance and changes in intermediate outcomes (i.e., sobriety motivation, mental health and physical health) that were mediated by psychological safety’s significant associations with hope, connection, and empowerment. In addition, we found a direct positive relationship between attending Phoenix events and physical health.

Participants’ high levels of psychological safety mediated the relationship between attending Phoenix events and experiencing connection. Prior work has shown social connection via engaging with others to increase self-efficacy and lead to improved health and quality of life (8, 11, 17). In addition, both mutual-help organizations and peer services emphasize how important forming connections are to support recovery (4). Our findings point to the potential benefit of providing psychologically safe environments for those with SUD, and community standards such as those utilized by The Phoenix to help facilitate a sense of psychological safety.

More broadly, the results highlight the importance of dynamic interactions between the individual and their social environment (8, 18, 19, 49). The Phoenix takes a community-based approach to support recovery, as it is not a treatment program. It provides accessible sober-active community events that aim to bolster personal growth and well-being through psychologically safe environments. This helps address the pressing need for easily accessible recovery support services (19). Phoenix attendance was positively associated with physical health improvement, and the full model explained 25–39% of the variance in the intermediate outcomes of sobriety motivation, mental health, and physical health through psychological safety, hope, connection, and empowerment. This aligns with previous research that has identified the importance of maintaining positive relationships in which individuals can connect with others without experiencing guilt or shame (8, 18, 49). As well, better mental health is associated with higher functioning during recovery (15). It is critical that recovery supports cultivate psychological safety in their respective environments and services (50), particularly as trauma-informed services (30).

Previous research found that feeling empowered was positively associated with self-rated health (14), supporting the positive association we found between empowerment and physical health. We also were not surprised to find a direct association between attendance and physical health, since most Phoenix events involved some type of physical activity (e.g., CrossFit, yoga, cycling). Physical activity is known to have considerable positive physical health benefits for individuals in recovery from substance use (51). However, it was surprising that a direct association was not also found for improvements in mental health, since physical activity also is known to have mental health benefits (e.g., helps to reduce stress, depression and anxiety) (52, 53). Perhaps this was due to unresolved trauma becoming more salient among some participants.

Individual characteristics may result in different perceptions of psychological safety (54). However, we did not find any differences in psychological safety ratings for any demographic groups. The only significant difference was that those new to recovery reported significantly greater improvements in hope than those in long-term recovery. This can be spurred through connections with other non-using peers, where people new to recovery begin to see that change and growth are possible which spurs hope (55). As individuals progress in their recovery, they experience a psychological change in their mind set, or a commitment to being ‘in recovery,’ along with a shift to a recovery-oriented identity (11) where they are receiving social support, and helping others helps sustain their own recovery (56). Sustaining hope helps maintain goal motivation for sobriety (13), such as we found in our model. These findings may also reflect the peer support inputs of The Phoenix as many volunteers who lead activities are themselves in recovery.

Key strengths of this study include that it has high external validity due to the program evaluation design. Findings show promising evidence in support of The Phoenix’s conceptual model, particularly for the foundational role of psychological safety. This is the first study to provide evidence for The Phoenix’s rapidly growing program (now serving over 500,000 members), highlighting its effectiveness and paving the way for further research in psychological safety as a driver of recovery. It is also the first study to examine psychological safety among individuals receiving services from a community-based recovery organization.

Important limitations to consider when interpreting the study findings include: (1) The retrospective nature of the data do not allow for causality or examination of which aspects may contribute more in the studied variables. (2) Events were offered by The Phoenix prior to the development of the model tested within this manuscript. Therefore, the model was not developed *a priori*, even though we were able to confirm relationships between key variables. (3) Only 13% of Phoenix members invited chose to complete a survey, likely leading to a degree of selection bias. It is possible that individuals who did not participate in the survey were different in important ways from those who did (e.g., felt less psychological safety, experienced less favorable outcomes, reinitiated substance use, did not enjoy activities and events). (4) Recovery status was not measured using a standardized assessment. (5) Measures used in the survey were chosen from validated self-report measures, when available, yet sometimes the number of items was reduced, or the wording was modified to better fit The Phoenix context. (6) Recall bias and self-serving bias pose threats to internal validity of the findings using thentests. (7) Due to the program evaluation design, there was no control group. (8) The sample was 73.5% White. Despite these limitations, this study has high external validity for The Phoenix as a supportive sober-active community for individuals in recovery from SUD.

Considerable efforts are needed to increase the number of Americans receiving support for SUD. This can be achieved by decreasing barriers to care by offering low-cost and free recovery support services that are accessible and destigmatizing. Supporting multiple recovery pathways, such as the approach taken by The Phoenix, enables people to select the path that fits them best (57). Mutual aid groups provide long-term support via social connections who are also in recovery (57). The Phoenix is particularly exciting because it does all these things, in addition to offering a novel, physically active recovery pathway that appeals to many who find traditional SUD treatment and mutual-help organizations not to be a good fit. The psychological safety measure used in this study should be further refined and tested at an organizational level and with other programs providing recovery support. Particular attention should be paid to the effect of interpersonal engagements during events with peers in recovery.

## 5 Conclusion

This study represents a new application of psychological safety—a concept predominately explored in corporate and healthcare environments—to the social sector, demonstrating its transformative potential for vulnerable populations, particularly those in recovery. We explored associations between attendance, psychological safety and recovery-related changes in a sample of Phoenix members, finding that psychological safety fully mediated the relationship between Phoenix attendance and increased hope, connection and empowerment which were then significantly related to improved motivation to stay sober, mental health and physical health. In addition, attending Phoenix events was directly associated with improvements in physical health. These findings using a theory-driven approach to evaluation support The Phoenix sober-active community conceptual model, framing the holistic recovery approach of The Phoenix that integrates a community of peers in recovery, or with a connection to the organization’s mission. By showing that psychological safety mediates key recovery-related outcomes, our findings provide actionable insights for community organizations seeking to improve engagement and support long-term recovery. Cultivation of psychologically safe environments may help addiction recovery programs foster hope and empowerment. Within such settings, offering accessible, engaging, and inclusive activities will likely reduce barriers to engagement while helping individuals form protective, pro-recovery relationships and social identities. However, additional research is needed to validate the measure of psychological safety used in this study. Future studies should also include a greater racial diversity among study participants. More research is also warranted exploring the role of psychological safety in SUD recovery pathways.

## Data Availability

The raw data supporting the conclusions of this article will be made available by the authors, without undue reservation.

## Footnotes

^1^ https://thephoenix.org/about-us/
