# Correction: Psychological safety mediates attendance and recovery-related outcomes within the Phoenix: a sober-active community

**DOI:** 10.3389/fpubh.2025.1712424

**Published:** 2025-10-13

**Authors:** Katie M. Heinrich, Brett Wyker, Beth Collinson, David Eddie, David Best, Jacquelyn Hillios

**Affiliations:** ^1^Department of Research and Evaluation, The Phoenix, Denver, CO, United States; ^2^Recovery Research Institute, Center for Addiction Medicine, Massachusetts General Hospital, Boston, MA, United States; ^3^Department of Psychiatry, Harvard Medical School, Boston, MA, United States; ^4^Centre for Addiction Recovery Research, Leeds Trinity University, Leeds, United Kingdom

**Keywords:** addiction recovery, substance use, alcohol use, peers, community-based organization, hope, empowerment, health

File **Supplementary Table 1** was erroneously published with the original version of this paper. The file has now been replaced.

The original version of this article has been updated.

