# Supplementary material for: Psychological safety mediates attendance and recovery-related outcomes within the Phoenix: a sober-active community
Source: Front Public Health. 2025 Mar 21;13:1458026. doi: 10.3389/fpubh.2025.1458026 (PMC11970132; doi:10.3389/fpubh.2025.1458026)
Supplement: Supplementary file 1 [file Table_1.DOCX]

**Table S1.** Comparison of original and modified measures for the study survey.

| **Original Questions** | **Original Answer Scale** | **Phoenix Questions Used** | **Answer Scale** |
| --- | --- | --- | --- |
| *Team Psychological Safety* [20] | | *Psychological Safety* | |
| N/A |  | To what extent do you feel welcomed at Phoenix? | 1= not at all; 2= not much; 3= somewhat; 4= a lot; 5= completely |
| Working with members of this team, my unique skills and talents are valued and utilized. | 1= very inaccurate; 7= very accurate | To what extent do you feel valued at Phoenix? |  |
| People on this team sometimes reject others for being different. | 1= very inaccurate; 7= very accurate [Reverse scored] | To what extent do you feel accepted at Phoenix? |  |
| Members of this team are able to bring up problems and tough issues. | 1= very inaccurate; 7= very accurate | How comfortable do you feel sharing your emotions with others at Phoenix? |  |
| If you make a mistake on this team, it is often held against you. | 1= very inaccurate; 7= very accurate [Reverse scored] | If you have relapsed or if you were to, how confident are you that you can/could return to Phoenix without being judged? |  |
| *Self Efficacy – modified 3 questions from the New General Self-Efficacy Scale* [38] | | *Self Efficacy* | |
| Q1. I will be able to achieve most of the goals that I set for myself. | 1 = strongly disagree; 2 = disagree; 3 = neither agree nor disagree; 4 = agree; 5 = strongly agree | Q1. I am able to achieve most of the goals that I have set for myself. | 1 = Not at all; 2 = Not much; 3 = Somewhat; 4 = A lot; 5 = Completely |
| Q2. When facing difficult tasks, I am certain that I will accomplish them. |  | Q2. When facing difficult tasks, I am certain that I will accomplish them. |  |
| Q5. I will be able to successfully overcome many challenges. |  | Q3. I am able to successfully overcome many challenges. |  |
